# Meetings of Societies

**Published:** 1935

**Authors:** 


### Meetings of Societies

### Royal Society of Medicine—Sections of Otology and Laryngology.

The combined summer meeting of these sections was held in Bristol on June 14th and 15th. On Friday, June 14th, Mr. E. A. Peters (President of the Section of Otology) took the chair at the University, and Mr. J. Morris read a paper on "**Characteristics of Electrical Deaf Aids.**"

He pointed out that there are three kinds of aids in use : (1) Portable aids, to be worn on the person ; consisting of carbon microphone, battery, ear-piece and connections. In this it was easy to amplify sound, but as a rule it was the higher tones that the patient needed to have amplified, while the lower range (about 150/300 double vibrations per second) were most readily amplified. As a consequence, in many cases the noise of the lower tones was so great that the patient was deafened, although the high tones, which represented the consonants and were essential to understand speech, were inadequately heard. (2) Semi-portable aids, needing a case, constructed on the principle of valve amplification : speech thus heard was little distorted, but weight and expense were against this form. (3) Fixed (valve) aids, for use in church and theatre, which could be made to help any form or degree of deafness, but were expensive and also limited by not being portable. It was difficult to prescribe an aid for a deaf person on the findings of a pure-tone test, since the rectifying properties of the human ear were not taken into account. The most important test for the public was that every reliable firm allowed trial at home of the aid, gratis or at nominal cost; no aid should ever be purchased unless it was found effective after such a trial.

Mr. W. M. Mollison read a paper on "**The Operative Treatment of Vertigo.**" It was first essential to make sure that there was not some lesion of the external or middle ear causing the vertigo; and a neurologist should eliminate the question of central nervous disease. In a group of patients with unilateral deafness and severe vertigo operation had definite value. The operation he advocated was to expose the external semi-circular canal and inject alcohol into the labyrinth. Of twenty-eight of his cases traced twenty-two were cured. Mr. Thacker Neville gave reports of three cases treated in the manner described, and Mr. A. J. M. Wright showed a case treated by injection of alcohol through the oval window.

The members were entertained at lunch in the Old Senate Room of the University by Dr. P. Watson-Williams. The Vice-Chancellor was present to welcome them to the University. Mr. Herbert Tilly reminded them that the last visit they had paid to Bristol was in 1911,* when Dr. Watson-Williams was President of the Laryngological Section, and expressed the thanks and pleasure of the members at this renewal of their former visit.

In the afternoon a Clinical Meeting was held at the Bristol Royal Infirmary, at the discussion following which Dr. P. Watson-Williams took the chair. Mr. A. J. Wright showed cases: (1) of congenital shortness of œsophagus; (2) thyroglossal cyst of tongue. Mr. E. Watson-Williams, two cases of brain abscess, with recovery; recurrent granuloma of larynx following mustard gas poisoning; tracheal obstruction from polypus of œsophagus; cervical tumour (? lymphosarcoma); gumma of larynx with double abductor paralysis. Miss S. B. Wigoder showed eight cases of cancer of nasal sinuses treated by radium implantation by Mr. Watson-Williams. Mr. G. R. Scarff: (1) nasal cartilage graft in a child; (2) vertigo with otorrhœa. Dr. P. Watson-Williams, chronic sinusitis with periodontal sepsis. Mr. Angell James: (1) aneurysm of internal carotid artery; (2) postcricoid carcinoma treated by radium; (3) leontiasis ossium; (4) frontal osteo-myelitis. Mr. E. Miles Atkinson, three cases of frontal sinusitis treated by Harmer's intubation

* *Bristol Medico-Chirurgical Journal,* vol. XXIX., p. 143.

method and two cases of cerebellar abscess. Mr. C. de W. Gibb, case of vascular tinnitus.

After the discussion the members were entertained at tea by the kind invitation of the President and Committee of the Royal Infirmary, and then proceeded to the new Chesterfield Nursing Home, where Mr. Wright provided cocktails and showed them round the new buildings.

In the meantime the ladies accompanying members had motored to Wells to see the Cathedral, and then to Cheddar, where Mrs. A. J. Wright entertained them at lunch. After this they visited the famous caves, and returned in time for the cocktail party. In the evening, after dinner at the Grand Spa Hotel, a concert was given by Miss Ursula Boase (songs and piano), Miss Joan Allen (violin), and Mr. Glyn Eastman (songs), and after this a dance was held. In the interval between dances Mr. "Charlie" Thomas entertained with lightning sketches and carefully-selected anecdotes.

---

On Saturday, June 15th, Mr. W. M. Mollison (President of Section of Laryngology) took the Chair. Mr. E. R. Chambers read a paper on "**Nasal Sinusitis and Infections of the Eyeball.** He pointed out that sinusitis was responsible for many cases of eye infection. In dealing with these no time must be wasted if permanent visual impairment was to be avoided. Such eye conditions were conjunctivitis, episcleritis , keratitis, irido-cyclitis, retinal hæmorrhages, particularly with sudden loss of central vision in young and robust patients, and papillœdema. While other sources of infection must not be neglected, in many of these cases a sinus infection was responsible, and when this was treated the hitherto intractable eye condition rapidly improved.

Mr. E. Watson-Williams followed with an account of the sinus conditions found in such cases. When there were obvious nasal symptoms often these indicated that the local resistance was good, and no remote damage followed. On the other hand, where there was little local reaction the possibilities of toxic absorption and remote damage were greater. Thus often in eye infections there were no local symptoms, signs or history to attract attention ; and even X-ray findings did not suffice to exclude sinusitis. Only by washing out such sinuses as experience showed were often diseased could a satisfactory examination be made ; the washings being cultured. If infection were found it was essential to drain the affected cells, to avoid recurrence of the eye trouble. Even very young children might suffer in this way. The results of treatment of the sinuses on the progress of the eye disease were very good.

Mr. Lionel Colledge read a paper on "**Tracheal Obstruction.**" This might be due to causes outside the trachea, tumours, enlarged glands, aneurysm, and most important of all, goitre and œsophageal cancer. In benign goitre operation to relieve pressure should be undertaken; in malignant goitre tracheotomy should be avoided. Diseases of the tracheal wall were rare. Syphilis, tubercle or new growth might be encountered. In the first, medical treatment gave good results without stenosis. In tubercle it might be necessary to excise the affected part of the trachea to obtain relief. Mr. F. C. Ormerod showed a number of pictures of tracheal obstruction from post-mortem specimens. He spoke particularly of bronchial carcinoma, and the route by which it invaded the trachea by penetrating the lung substance.

---

### Royal Society of Medicine—Orthopædic Section.

Provincial Meeting at Bath and Wessex Orthopædic Hospital, Bath, on Saturday, July 6th, at 2.15 p.m.

The Society was kindly favoured with the use of the Recreation Hut of the Royal United Hospital, which has recently been re-fitted and is admirably adapted for a demonstration of cases, such as that with which the meeting opened. Members attended from London, Birmingham, Bournemouth, etc., as well as members of the Bristol Medico-Chirurgical and the local British Medical Association. Mr. Todd, President of the Section, was in the Chair.

The clinical demonstration, which lasted from 2.15 p.m. to 5.0 p.m., was divided into sections:

1. Nerve cases of orthopædic interest, which were demonstrated by Dr. Ronald Gordon, the visiting Physician to the Orthopædic Hospital, and Miss Forrester-Brown, the senior surgeon. These included spastic cases treated by spa and operative methods; also various types of claw-feet, on which the President, Mr. Todd, kindly gave his opinion. An athetotic case was shown with admirable needle-work and type-script done by herself.

2. The Treatment of Infantile Paralysis, in connection with which Dr. Gordon reminded members that the latest research work has discredited the use of serum even in the pre-paralytic stage. Miss Forrester-Brown demonstrated operation results, including transplants for thenar paralysis and clawing ; and Mr. Bastow second surgeon, demonstrated the routine splinting used in the hospital.

3. Congenital Deformities, in connection with which Miss Forrester-Brown showed a series of club-feet, illustrating the difficulties in treatment which occur even when cases are treated regularly from birth ; also operations for irreducible congenital dislocations of the hip.

4. Splints and X-rays in connection with Hibb's method of fusion of quiescent tuberculous hips were on show, as well as numerous other pieces of apparatus, including two splints made of material provided by the Bristol Aeroplane Company, materials which promise well for lightness and speed of production in the future.

After this part of the meeting tea was provided in the Orthopædic Hospital Nurses' Home by the Matron, Miss Reid, and most of the visitors inspected the hospital.

